# Niaspan Attenuates the Adverse Effects of Bone Marrow Stromal Cell Treatment of Stroke in Type One Diabetic Rats

**DOI:** 10.1371/journal.pone.0081199

**Published:** 2013-11-26

**Authors:** Tao Yan, Xinchun Ye, Michael Chopp, Alex Zacharek, Ruizhuo Ning, Poornima Venkat, Cynthia Roberts, Mei Lu, Jieli Chen

**Affiliations:** 1 Neurology, Tianjin Medical University General Hospital, Tianjin Neurological Institute, Tianjin, China; 2 Neurology, Henry Ford Hospital, Detroit, Michigan, United States of America; 3 Neurology, The Affiliated Hospital of Xuzhou Medical College, Xuzhou, Jiangsu, China; 4 Physics, Oakland University, Rochester, Michigan, United States of America; 5 Public Health Sciences, Henry Ford Health System, Detroit, Michigan, United States of America; University of South Florida, United States of America

## Abstract

**Aims:**

Our previous studies have found that bone-marrow-stromal cells (BMSC) therapy improves functional recovery after stroke in non-diabetic rats while increases brain hemorrhage and induces arteriosclerosis-like changes in type-one-diabetic (T1DM) rats. Niaspan treatment of stroke increases vascular stabilization, decreases brain hemorrhage and blood-brain-barrier (BBB) leakage in T1DM rats. We therefore tested the hypothesis that combination therapy of BMSC with Niaspan attenuates the side effects of BMSC monotherapy in T1DM rats.

**Methods:**

T1DM-rats induced by streptozotocin were subjected to 2 hours of middle-cerebral-artery occlusion (MCAo) and treated with: 1) PBS; 2) BMSC (5×10^6^); 3) Niaspan (40 mg/kg) daily for 14 days; 4) BMSC (5×10^6^) +Niaspan (40 mg/kg, daily for 14 days) combination starting at 24 hours after MCAo. All rats were monitored for 14 days.

**Results:**

Combination BMSC+Niaspan treatment of T1DM-MCAo rats did not increase brain hemorrhage, and significantly decreased BBB leakage and vascular arteriosclerosis-like changes as well as decreased Angiogenin, matrix metalloproteinase 9 (MMP9) and ED1 expression in ischemic brain and internal-carotid-artery compared to non-treatment control and BMSC monotherapy animals.

**Conclusions:**

Combination therapy using BMSC with Niaspan decreases BBB leakage and cerebral arteriosclerosis-like changes. These beneficial effects may be attributed to the decreased expression of Angiogenin, MMP9 and ED1.

## Introduction

Diabetes mellitus (DM) is a leading health concern [1] that substantially elevates the risk of occurrence and recurrence of ischemic stroke [2]–[4]. Post stroke outcomes and fatality rates are significantly worse in the diabetic population [5], [6]. DM patients suffer from vascular damage and rapidly develop micro-vascular and macro-vascular diseases [1], [7]. Compared to the non-diabetic cases, DM patients face greater residual neurological and functional disability [3].

Previous studies have found that cell therapy with bone-marrow-stromal cells (BMSC) improves functional recovery after stroke in non-diabetic rats [8], [9]. BMSC have the ability to pass the blood brain barrier (BBB) and selectively target damaged brain, secrete growth factors [8]–[10], increase angiogenesis and synaptogenesis [11], as well as improve functional recovery after stroke and traumatic brain injury [9], [12], [13].

BMSC increase vascular stabilization and induce anti-inflammatory effects after stroke in non-diabetic rats [9], [11]. However, in type one diabetic mellitus (T1DM) rats, BMSC treatment when initiated 24h after stroke increases brain hemorrhage transformation, BBB leakage and induces cerebral arteriosclerosis-like changes which may be related to the increased expression of Angiogenin and ED1-positive macrophages in ischemic brain [14]. Niacin (nicotinic acid) increases High Density Lipoprotein (HDL) and improves endothelial function, reduces inflammation, increases plaque stability [15], and is widely used to treat arteriosclerosis [16]. Niaspan is a prolonged release formulation of niacin and is safely used in patients with diabetes [16]. Treatment of stroke with Niaspan significantly increases Angiopoietin-1 (Ang1) expression in ischemic brain which promotes vascular stabilization and maturation [17], decreases brain hemorrhage and BBB leakage in T1DM rats by reducing the pro-inflammatory factors [18], [19], and promotes vascular maturation and stabilization, which in concert improves functional outcome after stroke [18], [20]. Hence, we hypothesize that combination therapy of BMSC with Niaspan, attenuates BMSC-induced adverse side-effects in TIDM rats, and the present study investigated the effect of combination therapy of BMSC with Niaspan on BBB leakage, brain hemorrhage and cerebral arteriosclerosis-like changes in T1DM rats after stroke.

## Materials and Methods

All experimental procedures were carried out in accordance with the NIH Guide for the Care and Use of Laboratory Animals and approved by the Institutional Animal Care and Use Committee of Henry Ford Hospital (IACUC approval number: 999). All efforts were made to ameliorate suffering of animals.

### 2.1 Diabetes induction

Adult Male Wistar rats (225–250 g) purchased from Charles River (Wilmington, MA) were used. Diabetes was induced by a single intraperitoneal injection of streptozotocin (STZ, 60 mg/kg, Sigma Chemical Co., St. Louis, MO) to rat. The fasting blood glucose level was tested with caudal vein blood by using a glucose analyzer (Accu-Chek Compact System; Roche Diagnostics, Indianapolis, IN). Animals were subjected to middle cerebral artery occlusion (MCAo) 2 weeks after diabetes induction (fasting blood glucose >300 mg/dl) [21].

### 2.2 BMSC culture [9]

Bone marrow from normal male Wistar rats (n = 6/group) was isolated and cultured in Alpha-DMEM media with 20% fetal bovine serum and 1%penicillin streptomycin. Cells were maintained at 37°C in 5%CO_2_ and non-adherent cells were removed. BMSC were used within passage 4.

### 2.3 MCAo model and experiment groups

T1DM rats were anesthetized and transient (2h) MCAo was induced by using an intraluminal vascular occlusion [9]. Briefly, rats were anesthetized with 2% isoflurane in a jar for pre anesthetic, and spontaneously respired with 1.5% isoflurane in 2:1 N_2_O:O_2_ mixture using a facemask connected and regulated with a modified FLUOTEC 3 Vaporizer (Fraser Harlake, Orchard Park, NY 14127). Rectal temperature was maintained at 37°C throughout the surgical procedure using a feedback regulated water heating system. A 4–0 nylon suture with its tip rounded by heating near a flame was inserted into the external carotid artery (ECA) through a small puncture. The microsurgical clips were removed. The length of nylon suture, determined according to the animal's weight, was gently advanced from the ECA into the lumen of the internal carotid artery (ICA) until the suture blocked the origin of the middle cerebral artery (MCA). The nylon filament was retained inside the ICA for 2h and the neck incision was closed. The animals were moved to their cage to awaken. After 2h of MCAo, animals were re-anesthetized with isoflurane, and restoration of blood flow was performed by withdrawal of the filament until the tip cleared the lumen of the ECA. The incision was then closed. Rats were randomized and assigned to different groups and were treated starting 24h after MCAo with: 1) PBS (Phosphate Buffered Saline) as vehicle control (n = 8); 2) BMSC (5×10^6^) alone (n = 8) via tail vein injection; 3) Niaspan (40 mg/kg, Kos Pharmaceuticals, Inc. Cranbury. NJ; dissolved in saline) alone orally daily for 14 days (n = 10); 4) BMSC (5×10^6^) (intravenous injection) +Niaspan (40 mg/kg, orally daily for 14 days) combination treatment (n = 7). Rats were sacrificed at 14 days after MCAo, BBB leakage, brain hemorrhage and artery wall thickness were measured.

### 2.4 Functional tests

A battery of behavioral tests (modified neurological severity score, mNSS) and Foot-fault tests were performed prior to and at 1, 7 and 14 days after MCAo by an investigator who was blinded to the experimental groups.

**mNSS.** mNSS is a composite of motor, sensory, reflex and balance tests [22] **.** It includes motor testing like flexion of forelimb, hind limb and head movement when the animal is raised by the tail; ability to walk straight when placed on the ground; timed beam balance test; sensory that is visual and tactile tests and test of reflexes to sudden auditory noise, corneal reflex etc. During the tests, the inability to perform the task or a lack of reflex, scores 1 point. Hence, the higher the score the greater is the damage.

**Foot-fault test.** Foot fault test is used to assess motor impairment and coordination and placement dysfunctions of animal’s forelimbs [23]. The animal is placed on a metal wire grid that has openings and every time the animal incorrectly places its forelimb thereby falling through the opening, the event is recorded as a foot fault. The total number of steps (movement of each forelimb) the animal takes to cross the grid and the number of foot faults in that time span (usually 2 minutes) are recorded.

### 2.5 Lesion volume, histological and immunohistochemical assessment

All brains were fixed by transcardial perfusion with saline, followed by perfusion and immersion in 4%paraformaldehyde, and were then embedded in paraffin. Seven coronal sections of tissue were processed and stained with hematoxylin and eosin (H&E) for calculation of volume of cerebral infarction and presented as a percentage of the lesion compared with the contralateral hemisphere [24].

A standard block was obtained from the center of the lesion (bregma –1 mm∼+1 mm). A series of 6µm thick sections was cut from the block. Every 10^th^ coronal section for a total 5 sections was used for immunohistochemical staining.

Antibody against albumin (albumin-FITC, polyclonal, 1:500, Abcam, Cambridge, MA); α-smooth muscle actin (α-SMA, a SMC marker, mouse monoclonal IgG,

1:800; Dako); matrix metaloproteinase 9 (MMP9, 1:500, Santa Cruz Biotechnology, Santa Cruz, CA), ED1 (a mouse mAb against rat microglia/macrophages, monoclonal, 1:30; AbD Serotec Raleigh, NC) and Angiogenin (monoclonal, 1:500, Abcam Cambridge, MA) were employed. Control experiments consisted of staining brain coronal tissue sections as outlined above, but non-immune serum was substituted for the primary antibody. The immunostaining analysis was performed by an investigator blinded to the experimental groups.

**2.5.1 Brain Hemorrhage measurement.** Brain hemorrhage was measured by using H&E staining under light microscopy [17]. Measurements of brain hemorrhage included animals that died in the early stage after stroke. The percentage areas of petechial and gross hemorrhage were measured in each histological section and summed.

**2.5.2 Quantitation measurements of albumin, Angiogenin, MMP9 and ED1.** For quantitative measurements of Angiogenin, MMP9 and ED1, five slides from each brain, with each slide containing 8 fields of view from the ischemic border zone (IBZ) were digitized under a 20x objective (ED1, 40x objective )(Olympus BX40) using a 3-CCD color video camera (Sony DXC-970MD) interfaced with an MCID image analysis system (Imaging Research, St. Catharines, Canada). The sections of albumin immunostaining were observed and taken pictures with a fluorescence microscope (Zeiss Axiophot 2, Carl Zeiss MicroImaging, Inc.). The ischemic border zone is defined as the area surrounding the lesion. The data from 5 sections and 8 regions within each section were averaged to obtain a single value for 1 animal and presented as percentage of positive area of immunoreactive cells, respectively. Angiogenin and MMP9 expression in IBZ were measured and presented as percent of total scan area. The numbers of ED1-immunoreactive cells were counted. The total number of ED1 positive cells per millimeter squared are presented. Angiogenin, MMP9 and ED1 expression in the bilateral ICA of each section were measured and presented as percent of luminal area of ICA. Data were analyzed in a blinded manner.

**2.5.3 α-SMA positive coated vessel density measurement.** α-SMA immunoreactivity was employed as a marker to identify arteries [25]. The density of α-SMA-stained vessels was analyzed with regard to small and large vessels (>10μm diameter) in the IBZ. The arterial wall thicknesses were measured in the ten largest arteries according to the arterial diameter. In addition, the total number of occluded arteries in the ipsilateral hemisphere was counted.

**2.5.4 Double immunohistochemical staining.** FITC (Calbiochem) and cyanine-5.18 (CY5, Jackson Immunoresearch) were used for double-label immunoreactivity. Each coronal section was first treated with the primary anti-Angiogenin or anti-ED1 antibody with Cy5, and then followed by ED1/MMP9 with FITC. Control experiments consisted of staining brain coronal tissue sections as outlined above, but use non-immune serum for the primary antibody.

### 2.6 Statistical Analysis

Chi-square test was used to test proportional mortality difference among groups. Generalized Estimation Equations (GEE) were used to test the functional recovery measured from a set of behavioral tests among the groups [26]. One-way analysis of variance (ANOVA) was used for the immunostaining data. Data analysis started testing the overall group effect, followed by subgroup analysis. All data were measured by investigators blinded to the experiments.

## Results

### Combination BMSC and Niaspan treatment of stroke did not improve functional outcome after stroke in T1DM rats, but significantly decreases BBB leakage in the ischemic brain after stroke in T1DM rats

**Figure 1:**
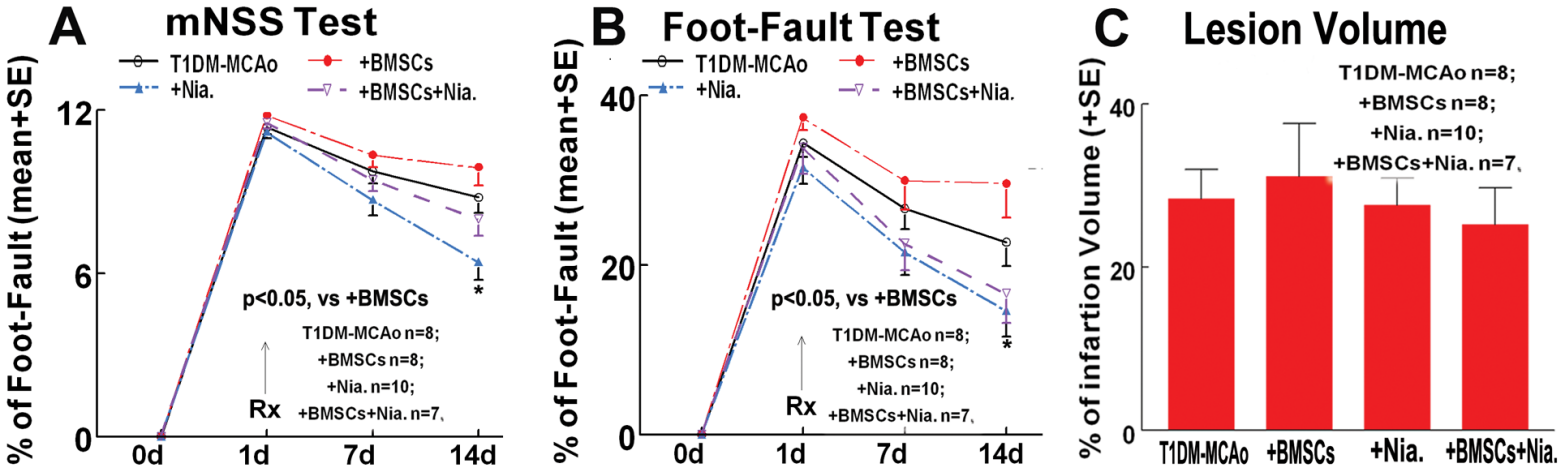
Functional tests and lesion volume measurements. Combination treatment did not improve functional outcome measured by mNSS test (A) and foot-fault test (B), and did not decrease lesion volume (C) after stroke when compared with T1DM control or BMSC monotherapy groups.

To test whether combination treatment attenuates BMSC induced worse functional outcome after stroke in T1DM rats, a battery functional tests were performed as shown in Figures 1A-B. Consistent with previous studies, Niaspan treatment of stroke in T1DM rats significantly improves functional outcome [17]. However, we did not find that combination treatment significantly improved functional outcome after stroke in T1DM control rats when compared to T1DM control or BMSC monotherapy groups. Although, there is no significant difference (p>0.05) in functional outcome when comparing the combination treatment to T1DM-control or BMSC monotherapy, combination treatment results in a trend (p = 0.07) of decreased mortality rate (22%) compared with BMSC monotherapy (50%) animals. Mortality rate in T1DM-MCAo: 25%; +Niaspan monotherapy: 14.3%; BMSC monotherapy: 50%; combination therapy: 22%. In addition, combination BMSC and Niaspan treatment of stroke in T1DM initiated at 24h after MCAo did not decrease lesion volume (Figure 1C).

**Figure 2:**
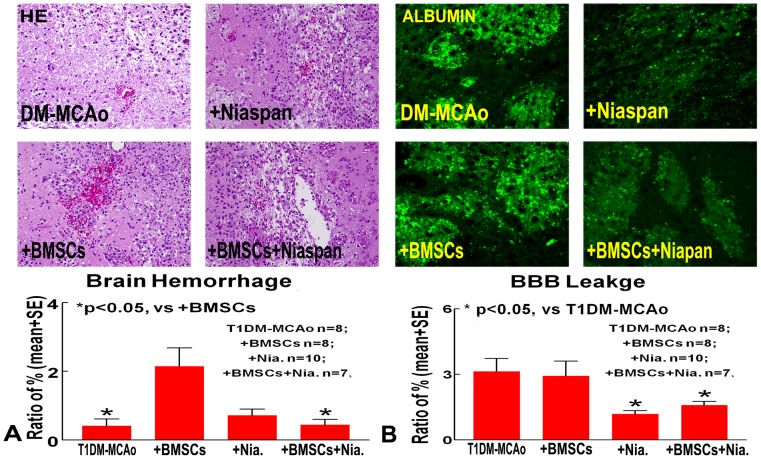
Brain hemorrhage and BBB leakage measurements. Combination treatment of stroke with BMSC+Niaspan did not decrease brain hemorrhage transformation with HE staining (A) compared with BMSC treatment group, while Niaspan and combination treatment significantly decreased BBB leakage as identified by albumin staining (B) compared to T1DM-MCAo control.

To test whether combination treatment of stroke in T1DM rats regulates BBB leakage, albumin staining was performed. Albumin infiltration into the ischemic brain is a marker of BBB leakage [27]. The data show that combination BMSC and Niaspan treatment of stroke in T1DM initiated at 24h after MCAo did not decrease brain hemorrhage transformation (Figure 2A), but decreased BBB leakage compared with BMSC monotherapy in T1DM animals (Figure 2B) identified by albumin staining (p<0.05).

### Combination BMSC and Niaspan treatment of stroke decreases cerebral arteriosclerosis-like changes identified by cerebral arterial wall thickness and number of occluded arteries

**Figure 3:**
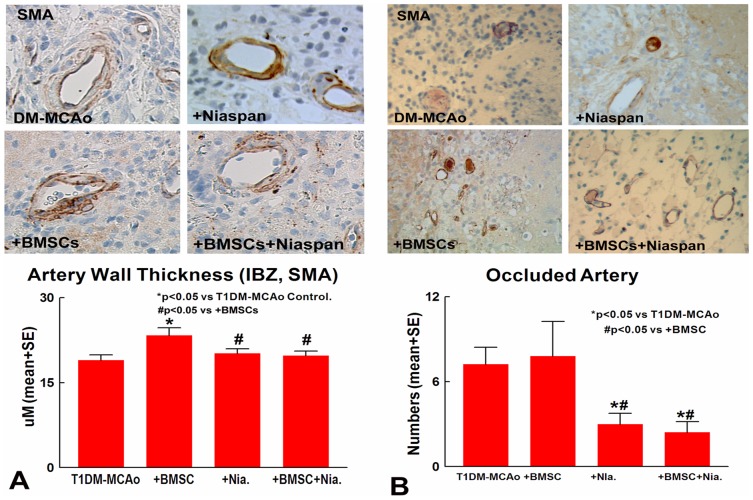
Cerebral artery changes measurement. Treatment of stroke with BMSC significantly increased arterial wall thickness and the number of occluded arteries in the ischemic brain. Niaspan and combination BMSC+Niaspan treatment exhibits significantly decreased arterial wall thickness (A) and number of occluded arteries (B) in the ischemic brain in T1DM rats compared to BMSC monotherapy treatment or T1DM-MCAo control groups.

Our previous studies have found that BMSC treatment of stroke in T1DM rats significantly induced arteriosclerosis-like changes [14]. To test whether combination treatment attenuates BMSC induced arteriosclerosis-like changes, cerebral arterial wall thickness and number of occluded arteries were measured in the ischemic brain. Treatment of stroke with BMSC significantly increased arterial wall thickness and the number of occluded arteries in the ischemic brain. Figure 3 indicates that Niaspan and combination of BMSC and Niaspan treatment exhibit significantly decreased arterial wall thickness (Figure 3A) and number of occluded arteries (Figure 3B) in the ischemic brain of T1DM rats compared to BMSC monotherapy treatment (p<0.05) or T1DM-MCAo control groups (p<0.05). Our data suggest that Niaspan and BMSC combination treatment significantly attenuates the adverse vascular effects (arteriosclerosis-like changes) of BMSC monotherapy in T1DM animals.

### Combination therapy of BMSC and Niaspan attenuates BMSC induced Angiogenin, MMP9 and ED1 expression in the IBZ and in the ICA

**Figure 4:**
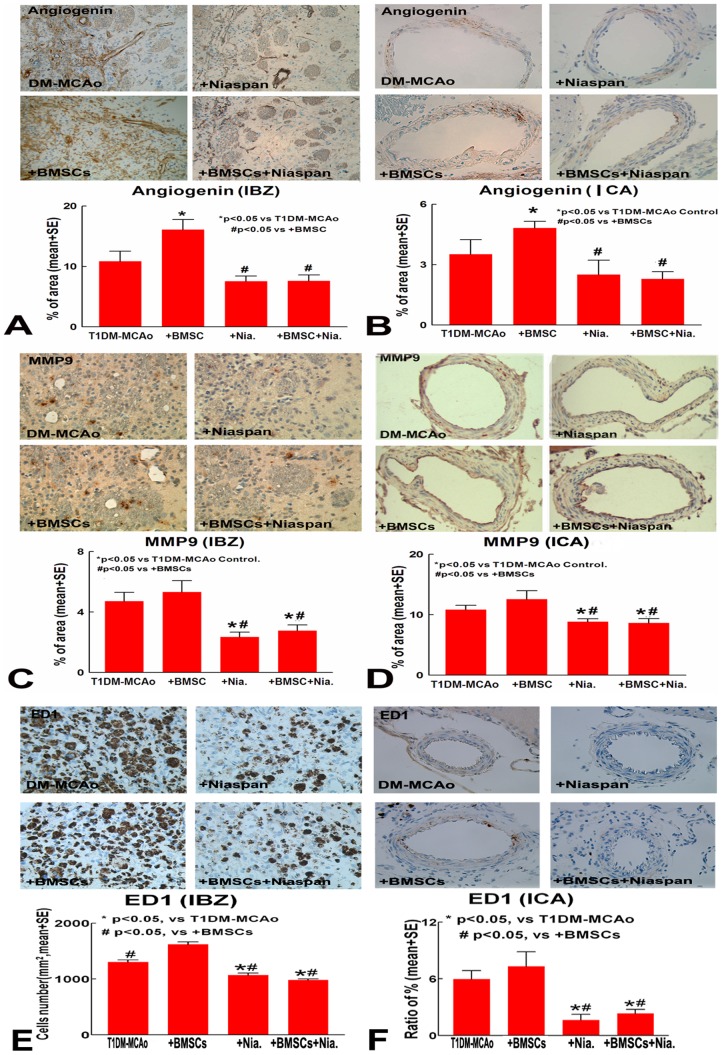
Angiogenin, MMP9 and ED1 expression. Treatment of stroke with BMSC significantly increased angiogenin expression in IBZ (A) and arterial wall (B) of T1DM rats; Niaspan and combination BMSC with Niaspan treatment significantly attenuated BMSC induced Angiogenin (A,B), MMP9 (C,D) and ED1(E,F) expression in the ischemic brain and in the ICA compared to T1DM-MCAo control and BMSC monotherapy treatment group.

To test the mechanism of combination treatment reduction of BBB leakage and arteriosclerosis-like changes, Angiogenin, MMP9 and ED1 expression were measured in the IBZ and ICA. Consistent with our previous studies, treatment with BMSC alone significantly increased Angiogenin (Figure 4A-B), MMP9 (Figure 4C-D) and ED1(Figure 4E-F) expression in the IBZ and in the ICA. However, Niaspan and combination of BMSC+Niaspan treatment significantly attenuated BMSC induced Angiogenin, MMP9 and ED1 expression in the ischemic brain and in the ICA compared to T1DM-MCAo control and BMSC monotherapy treatment group (p<0.05 vs. T1DM-MCAo control; p<0.05 vs. BMSC monotherapy treatment group). These data suggest that Niaspan treatment attenuates BMSC treatment induced inflammatory effects in the ischemic brain after stroke in the T1DM rats.

### MMP9 positive cells are co-localized with ED1 and Angiogenin positive cells

**Figure 5:**
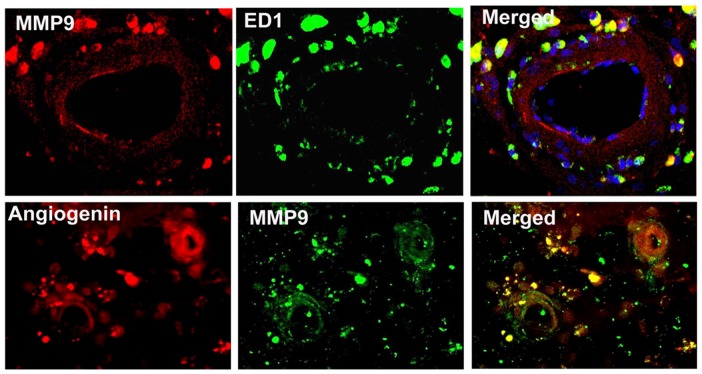
MMP9/ED1 and Angiogenin double immunostaining. Double immunostaining shows that MMP9 positive cells are co-localized with ED1 (macrophage cell marker) and Angiogenin positive cells.

To specifically identify MMP-reactive cells co-localized with macrophages, double immunostaining of MMP9/ED1 and Angiogenin/MMP9 was performed. Figure 5 shows that MMP9 positive cells are co-localized with ED1 (macrophage cell marker) and Angiogenin positive cells.

## Discussion

In this study we have demonstrated that treatment of stroke with combination of BMSC and Niaspan attenuates vascular adverse effects, such as BBB leakage and atherosclerotic-like changes induced by BMSC monotherapy on T1DM rats after stroke and has a trend of decreased early mortality (p = 0.07) in T1DM rats..

Early mortality after stroke is correlated with brain hemorrhage formation and BBB leakage [17]. Treatment of stroke with BMSC initiated at 24h after MCAo, increases BBB leakage and brain hemorrhage which may contribute to the decrement of functional outcome after stroke in T1DM rats. In this study, combination treatment with Niaspan+BMSC did not upregulate BMSC induced brain hemorrhage formation, but significantly attenuated BBB leakage in the ischemic brain when compared to T1DM-MCAo control. Although the combination treatment has a trend of decreased early mortality (p = 0.07) in T1DM rats, it does not significantly improve functional outcome. The combination treatment failure to improve functional outcome may be attributed to the small sample size employed and the early sacrifice time. In addition, the positive Niaspan effects may simply cancel the negative effects of BMSC for the presently employed duration of treatment. Long term treatment with combination of BMSC and Niaspan may be able to improve functional outcome. Therefore, further studies with additional animals and long term observation are needed.

Diabetes upregulates MMP9 expression and activity, which may contribute to increased brain hemorrhage and BBB leakage after stroke [28]. Activation of extracellular components like MMPs can cause damage to the basal lamina, alter microvascular endothelial function and trigger disruption of the BBB thereby increasing its permeability [29], [30]. MMP-9 with its proteolytic activity attacks extracellular matrix (ECM) components including collagen, elastin and regulatory factors of angiogenesis, growth and apoptosis [31]. MMP9 has been implicated in increasing neurological deficit, infarct volume and stroke severity [32], [33]. Within the first 48h after acute stroke, MMP9 overproduction is reported and elevated levels of MMP9 are detrimental to neurological recovery [32]. Angiogenin is a small protein with ribonucleolytic activity and is a potent angiogenic factor implicated in tumor growth [34], [35]. Angiogenin degrades the basement membrane and the ECM thereby acting as a stimulus that promotes the invasion and migration of endothelial cells into the surrounding tissue towards the source of stimulus [36]–[38]. Significantly increased Angiogenin expression has been correlated with many pathological conditions and disease severities like heart failure [39], pathogenesis of myocardial ischemia [37] and diabetic retinopathy [40]. In patients with peripheral artery occlusion, elevated Angiogenin levels in the circulation marks disease severity associated with total vessel occlusion, endothelial damage, tissue repair and adaptive revascularization [41].

Increased serum Angiogenin levels, have been reported among diabetic youngsters [42] and is a potential marker for micro vascular complications [43]. In T1DM rats, Angiogenin and MMP9 expression significantly increased in the ischemic brain. BMSC treatment alone enhanced Angiogenin, MMP9 and ED1 expression in the ischemic area which have pro-inflammatory effects [14], [44]. Inflammation is critical to the pathogenesis of tissue damage in cerebral infarction [45], [46]. Previous studies found that BMSC induced immunosuppressant effects in non-diabetic populations [47]–[49]. However, BMSC treatment in T1DM rats significantly increased vascular inflammatory response identified by increased Angiogenin, MMP9 and ED1 expression as well as increased arteriosclerosis-like vascular structure changes.

Recent studies and clinical trials have investigated the effects of Niacin and Niaspan on cardiovascular diseases and found that treatment with Niacin alone or its use in combination with statins improves lipid profile and reduces coronary arteriosclerosis by mediating anti-inflammatory events [50]–[54]. Niacin significantly reduced carotid arteriosclerosis, substantially increased HDL and decreased LDL [55] and exhibits anti-inflammatory and anti-arteriosclerosis effects [56]. We found that combination treatment of stroke in T1DM rats significantly decreased MMP9 and Angiogenin expression compared to BMSC monotherapy or T1DM-control groups.

From double immunostaining, MMP9 positive cells are co-localized with ED1 and Angiogenin positive cells. ED1 is a macrophage cell marker and a known inflammatory factor that can cause arteriosclerosis and secretion of inflammatory angiogenic factors [57]. Neuroinflammatory responses activate glial cells by triggering inflammatory mediators like cytokines, prostaglandins and free radicals that can act on the immune system [58]. BMSC noticeably increase Angiogenin expression in the arterial wall and ischemic brain which correlate with arteriosclerosis-like changes [14]. Treatment with Niaspan alone and in combination with BMSC resulted in significantly attenuated BMSC induced Angiogenin and MMP9 expression in the ischemic brain and in the ICA compared to T1DM-MCAo control and BMSC monotherapy treatment group. This is consistent with other findings that suggest that inhibition of MMP9 attenuates neuroinflammation and promotes recovery after stroke [59]. Therefore, attenuation of BMSC induced cerebral arteriosclerosis-like changes by Niaspan may derive from the decrease in ED1 and down-regulation of Angiogenin and MMP9 expression in T1DM rats.

## Conclusion

Combination therapy of BMSC and Niaspan attenuates the adverse effects of BMSC in T1DM animals. Niaspan monotherapy and combination of Niaspan with BMSC treatment of stroke substantially decrease Angiogenin, MMP9 and ED1 expression compared with BMSC monotherapy which may contribute to the attenuated BBB leakage and cerebral arteriosclerosis-like changes in the ischemic brain in T1DM rats after stroke.
